# *Candida krusei* M4CK Produces a Bioemulsifier That Acts on Melaleuca Essential Oil and Aids in Its Antibacterial and Antibiofilm Activity

**DOI:** 10.3390/antibiotics12121686

**Published:** 2023-11-30

**Authors:** Jéssica Mayra Mendes Araujo, Joveliane Melo Monteiro, Douglas Henrique dos Santos Silva, Amanda Karoline Veira, Maria Raimunda Chagas Silva, Fernanda Avelino Ferraz, Fábio H. Ramos Braga, Ezequias Pessoa de Siqueira, Andrea de Souza Monteiro

**Affiliations:** 1 Rede de Biodiversidade e Biotecnologia da Amazônia Legal, BIONORTE, Saint Louis 65055-310, Brazil; jessicamendesaraujo1@hotmail.com (J.M.M.A.); joveliane-12@hotmail.com (J.M.M.); 2 Laboratório de Microbiologia Aplicada, Universidade CEUMA, Saint Louis 65075-120, Brazil; douglasbmdh@gmail.com (D.H.d.S.S.); amandavieiraalencar@gmail.com (A.K.V.); nanda_ferras@hotmail.com (F.A.F.); 3 Laboratório de Ciências do Ambiente, Universidade CEUMA, Saint Louis 65075-120, Brazil; marirah@gmail.com (M.R.C.S.); fabiobraga.ma@gmail.com (F.H.R.B.); 4 Laboratório de Química de Produtos Naturais, Centro de Pesquisas René Rachou Fundação Oswaldo Cruz, Belo Horizonte 30190-002, Brazil; ezequias.siqueira@gmail.com

**Keywords:** *Candida krusei*, essential oil, bioemulsifier, antimicrobial activity

## Abstract

Surface-active compounds (SACs) of microbial origin are an active group of biomolecules with potential use in the formulation of emulsions. In this sense, the present study aimed to isolate and select yeasts from fruits that could produce SACs for essential oil emulsions. The *Candida krusei* M4CK was isolated from the *Byrsonima crassifolia* fruit to make SACs. This emulsification activity (E_24_) was equal to or greater 50% in all carbon sources, such as olive oil, sunflower oil, kerosene, hexane, and hexadecane. E_24_ followed exponential growth according to the growth phase. The stability of emulsions was maintained over a wide range of temperatures, pH, and salinity. The OMBE4CK (melaleuca essential oil emulsion) had better and more significant inhibitory potential for biofilm reduction formation. In addition, bioemulsifier BE4CK alone on *Escherichia coli* and *Pseudomonas aeruginosa* biofilm showed few effective results, while there was a significant eradication for *Staphylococcus aureus* biofilms. The biofilms formed by *S. aureus* were eradicated in all concentrations of OMBE4CK. At the same time, the preformed biofilm by *E. coli* and *P. aeruginosa* were removed entirely at concentrations of 25 mg/mL, 12.5 mg/mL, and 6.25 mg/mL. The results show that the bioemulsifier BE4CK may represent a new potential for antibiofilm application.

## 1. Introduction

In recent years, the production of microbial Surface-Active Compounds (SACs) has attracted attention due to its diverse applicability, such as the ability to play an essential role in the solubility of insoluble compounds in water, in the binding of heavy metals, in the desorption of contaminants, in bacterial pathogenesis, and in adhesion and cell aggregation [1,2,3,4]. In addition to these characteristics, SACs have several advantages over synthetic surfactants; for example, low toxicity, lower critical micellar concentration (CMC), excellent biodegradability, and ecological acceptability [5]. The main microbial SACs are biosurfactants and bioemulsifiers; these have different chemical compositions and considerable biological activities [3,4,6].

SACs can exhibit antibacterial, antifungal, antiviral, and antitumor activities [1,7,8]. The properties of anti-adhesive and antibiofilm activities are also essential, as they can inhibit adhesion and colonization by pathogenic microorganisms and remove pre-formed biofilms on silicone rubber and other biomedical instruments [9].

Another relevant factor about SACs is their production by various microorganisms, including bacteria, yeasts, and filamentous fungi [10,11]. Yeasts have been constantly used as leavening agents for SACs, as they are generally considered to have a safe (GRAS) status. Organisms characterized by GRAS status do not pose risks of toxicity or pathogenicity, which allows for their available applications in the pharmaceutical and food industries [11,12].

In the current scenario, yeasts have been considered the future molecules, as they have a GRAS *status* and synthesize SACs that act both in bioremediation and the biological field [6]. Several studies have shown that surfactant compounds produced by these microorganisms have potential biomedical applications since they exhibit antibiofilm, antibacterial, anticancer, larvicidal, and immunomodulatory activities [13].

Oil-in-water emulsions are efficient delivery systems for oily compounds, such as essential oils, dispersing the lipid phase as a colloidal dispersion [12,14]. Emulsifying agents prevent droplet coalescence by decreasing interfacial tension. SACs have attracted significant interest in elaborating emulsions and nanoemulsions, considering several physical and chemical characteristics [14]. They can be used to reduce volatility, increase stability, increase bioactivity, and extend the shelf life of essential oils for different applications, such as food, cosmetics, and the preparation of pharmaceutical formulations [14]. 

Previously, it was demonstrated that yeasts isolated from environments can produce emulsifiers that present great stability of emulsions [6]. Considering their applicability in biotechnology, studying the diversity of yeasts associated with fruits and the study of their molecules with emulsifying potential becomes enjoyable. They think that emulsions for essential oils are formulations of interest in the pharmaceutical industry and the preparation of cosmetics, among other applications. This study aims to isolate yeast biopolymers to prepare essential oil emulsions and quantify the synergistic effect against bacteria and microbial biofilms.

## 2. Results

### 2.1. Isolation of Yeasts Producing Surface-Active Compounds

In the present study, yeasts (*n* = 13) were isolated from fruits *Anacardium occidentale*, *Byrsonima crassifolia*, *Citrus reticulata*, and *Platonia insignis* (Table 1). MALDI-QTOF-MS identified the yeast strains as *Candida metapsilosis* and *Candida krusei.*

The isolated yeasts were inoculated separately in minimal minerals containing sunflower oil and glycerol at concentrations of 2%. Table 1 demonstrates the best source of carbon and energy for growth.

### 2.2. Screening and Identification of SAC-Producing Microorganisms

#### 2.2.1. Determination of Emulsifying Activity (E_24_)

This study aimed to investigate the emulsification properties of the surface-active compound, so the E_24_ test was mainly used to determine the presence of SACs in the samples. 

The most efficient yeast strain in producing surface-active compounds with emulsifying activity for several carbon sources was isolated from the fruit *Byrsonima crassifolia,* popularly known as murici and identified as *Candida krusei* M4CK. E_24_ was determined, and the values were indicated (Supplementary Table S1). The SACs produced by *C. krusei* M4CK were the only ones that showed emulsifying activity in all carbon sources.

**Figure 1:**
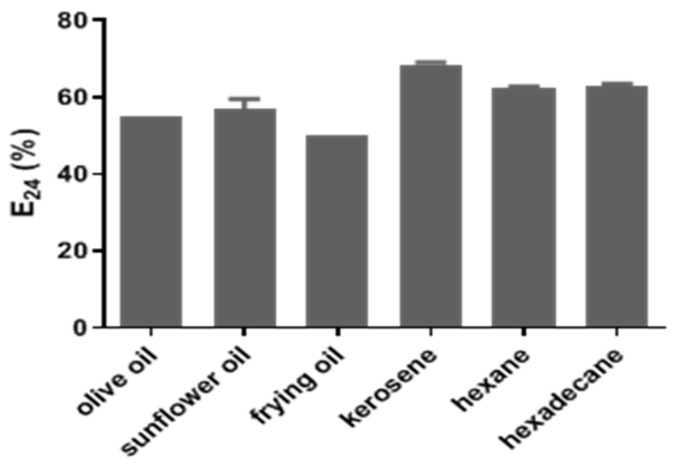
Emulsifying activity (E_24_) presented by SACs produced by *Candida krusei* M4CK in different hydrophobic compounds.

The emulsification activity of SACs contained in the supernatant of MM medium with glycerol as a carbon source was appreciable. The SACs, called *Candida krusei* M4CK, showed E_24_ values of 55%, 56.75%, 50%, 68.5%, 63.6%, and 63.95% for olive oil, sunflower oil, frying oil (used), kerosene, hexane, and hexadecane, respectively (Figure 1). The values of the final surface tension (mN/m) in the medium after cell growth were determined using a tensiometer and ranged from 44 to 62 mN/m (Supplementary Table S2).

#### 2.2.2. Determination of Surface Tension

The production of biosurfactants and bioemulsifiers by yeast isolates can be identified indirectly through surface activity and emulsifying activity. In this study, it was observed that the compound BE4CK showed a reduction in surface tension from 72 mN/m to 44 mN/m. This result demonstrated no reduction in surface tension to values below 30 mN/m. For this reason, it was characterized as a bioemulsifier (BE4CK).

### 2.3. Determination of Chemical Composition

Analyzes of the chemical composition of SAC (BE4CK) revealed the presence of carbohydrates (46%), protein (38%), and a lower amount of lipid parts (3%). GC characterized the BE4CK that indicated in its structure; the major fatty acid was 9-octadecenoic acid, (E)-(58.43%), followed by methyl stearate (16.72%) and hexadecanoic acid (13.44%). Moreover, the monosaccharides were mannose (84.26% and glucose (15.74%). 

### 2.4. Growth Kinetics

**Figure 2:**
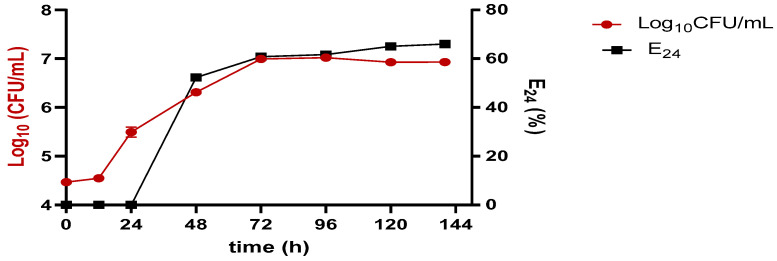
Kinetics of cell growth by *Candida krusei* M4CK and emulsifying activity by BE4CK. BE4CK: Bioemulsifier; CFU/mL: colony-forming unit per milliliters; E_24_: emulsifier activity.

*C. krusei* M4CK growth and emulsifying activity in the mineral medium supplemented with 2% glycerol are shown in Figure 2. For the strain, bioemulsifier and E_24_ production follow an exponential growth phase growth. Bioemulsifier production continues even when microbial growth ceases. About 52.3% of E_24_ activity in kerosene was obtained in the exponential growth phase in the first 40 h; after this period, E_24_ activity increased, reaching a percentage ≥ 60% in 60 h–144 h. These results indicate that the emulsification activity is partially associated with growth, as the peaks of bioemulsifier production did not coincide. The highest stability and emulsification activity was achieved at 144 h (Figure 2).

### 2.5. Evaluation of Bioemulsifier Stability

#### 2.5.1. Effect of Temperature on the Emulsifying Activity

**Figure 3:**
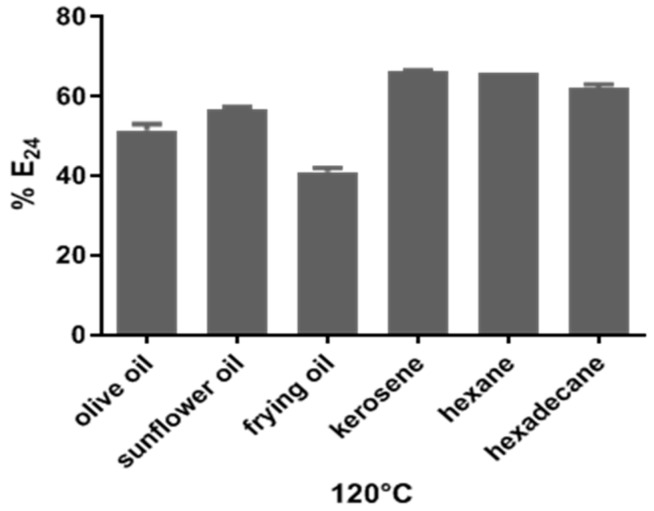
Emulsifying activity (E_24_) of BE4CK after heating to 120 °C. Values indicate the mean of duplicate values and error bars represent the standard deviation (SD).

After being subjected to the autoclaving process at 120 °C for 15 min, BE4CK showed stability and maintenance of emulsifying properties since it emulsified all the hydrophobic sources (Figure 3). The E_24_ of the bioemulsifier was ≥50% in olive and sunflower oil, while for kerosene, hexane, and hexadecane hydrocarbons, it was greater than 60%. For frying oil (E_24_ of 40%), there was a 10% reduction in the primary emulsifying activity (Figure 3).

#### 2.5.2. Effect of pH on the Emulsifying Activity

**Figure 4:**
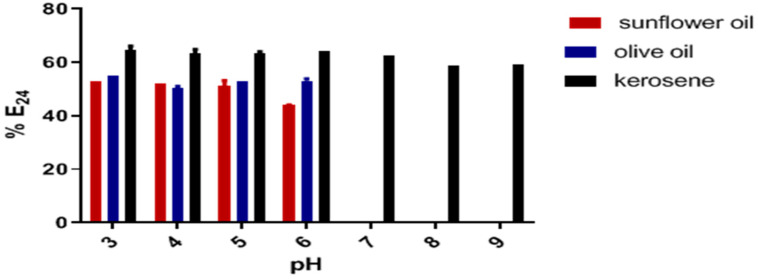
Emulsifying activity (E_24_) of BE4CK in different pH ranges. Values indicate the mean of duplicate values, while error bars represent standard deviation in pH: hydrogen potential.

The SAC produced by *C. krusei* showed emulsifying activity for different sources at acidic and basic pH (Figure 4). At acidic pHs (3–6), emulsification activity was ≥44% for all substrates (sunflower, olive, and kerosene), while at basic pHs, emulsification was characterized only for kerosene hydrocarbons. In this source, there was no marked variation in E_24_ in the pH ranges of 3 to 9. From pH eight onwards, the bioemulsifier produced by the *C. krusei* M4CK strain could emulsify only kerosene (Figure 4). In the range of pH 10, there was no emulsification stability for any of the sources.

#### 2.5.3. Effect of Salinity on Emulsifying Activity

**Figure 5:**
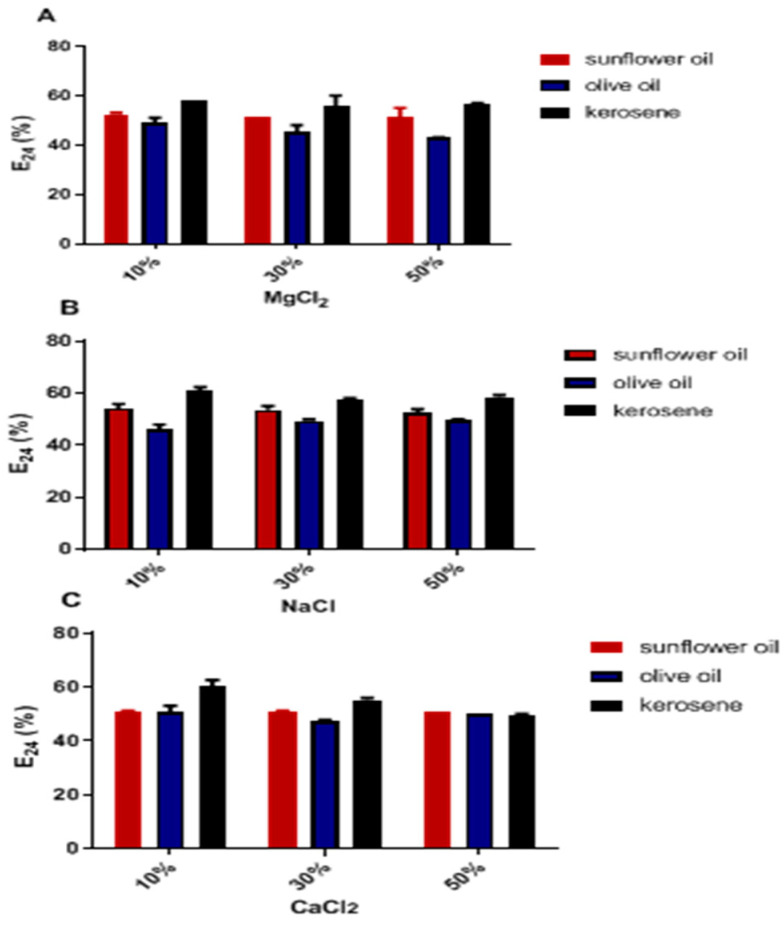
Stability of emulsifying activity in different salt concentrations. (**A**) MgCl_2_: magnesium chloride; (**B**) NaCl: sodium chloride; (**C**) CaCl_2_: calcium chloride. Values are three independent experiments’ mean ± standard deviation (SD).

It was observed that the E_24_ of the bioemulsifier in concentrations (10%, 30%, and 50%) of magnesium chloride (MgCl_2_), sodium chloride (NaCl), and calcium chloride (CaCl_2_) had contents ranging from 40 to 55% in vegetable oils (sunflower and olive) and for kerosene, a percentage ≥ 56%. The highest E_24_ value was obtained with kerosene, while the lowest was registered with olive oil (Figure 5).

The stability of the emulsifying activity (E_24_) was maintained at concentrations of 50% of the salts. It was also verified that as the salinity increased, there was a slight reduction in the emulsification rate. However, changes in salt concentrations do not influence E_24_ values (Figure 5).

### 2.6. Assessment of Bioemulsifier Cytotoxicity

**Figure 6:**
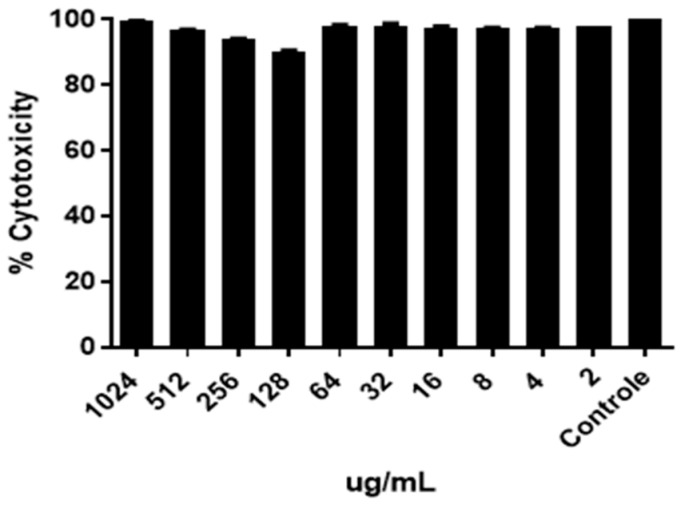
Evaluation of the cytotoxicity of the bioemulsifier BE4CK (1024 µg/mL–2 µg/mL) in VERO cells.

By the toxicity test, BE4CK showed no toxicity in VERO cells at concentrations of 1024 µg/mL–2 µg/mL, and the percentage of cell viability was ≥88.89% (Figure 6).

### 2.7. Determination of Antimicrobial Activity

When evaluating antimicrobial activity through the agar diffusion assay (Supplementary Figure S1), it was found that isolates of *E. coli* ATCC 25922, *E. faecalis* ATCC 35218, *P. aeruginosa* ATCC 27853, and *S. aureus* ATCC 25923 were significantly sensitive to the essential oil of melaleuca considering *p* < 0.05 (Table 2). In the case of BE4CK, the growth of microorganisms was not affected. The emulsion OMBE4CK showed an increase in the growth inhibition zone, corresponding to 34.5 mm for *E. coli* ATCC 25922. In comparison, melaleuca oil alone formed an inhibition zone of 28.5 mm for this microorganism. Thus, the compound combination showed antimicrobial activity, making *E. coli* ATCC 25922 more sensitive (Table 2 and Supplementary Figure S1).

Unlike BE4CK, the OMBE4CK emulsion and melaleuca essential oil showed significant inhibitory activity against *E. coli* ATCC 25922, *E. faecalis* ATCC 35218, and *S. aureus* ATCC 25923. The emulsion could not inhibit the growth of *P. aeruginosa* ATCC 27853.

### 2.8. Determination of Antibiofilm Activity

#### 2.8.1. Inhibition of Biofilm Formation on Polystyrene

**Figure 7:**
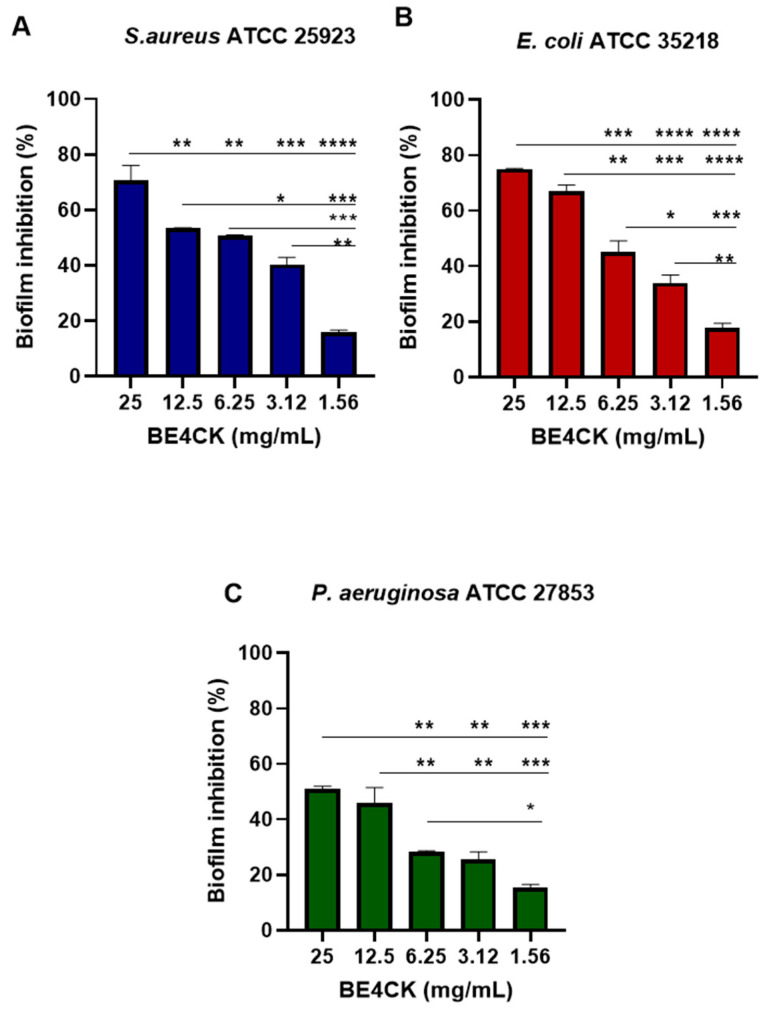
BE4CK inhibitory activity on biofilm formation by microorganisms. (**A**) *S. aureus* ATCC 25923; (**B**) *E. coli* ATCC 25922; (**C**) *P. aeruginosa* ATCC 27853. Values are three independent experiments’ mean ± standard deviation (SD). * (*p* < 0.05), ** (*p* < 0.001), *** (*p* < 0.001), **** (*p* < 0.0001)—a significant difference in relation to concentrations.

The antibiofilm potential of the bioemulsifier (BE4CK) produced by *C. krusei* M4CK was determined by its ability to impair and inhibit biofilm formation by *S. aureus* ATCC 25923, *E. coli* ATCC 25922, and *P. aeruginosa* ATCC 27853. The compound showed a dose-dependent effect, with the most significant activity at 25 mg/mL concentrations (*p* < 0.001). Inhibition percentages for these three isolates ranged from 51 to 75% at a concentration of 25 mg/mL of the bioemulsifier (Figure 7A–C). Applied at this concentration, BE4CK showed significant (*p* < 0.001) inhibitory potential against *S. aureus* ATCC 25923 (71%), *E. coli* ATCC 25922 (75%), and *P. aeruginosa ATCC* 27853 (51%). Even at lower concentrations (1.56 mg/L–3.12 mg/L) it was effective against *S. aureus* ATCC 25923 (16–40%), *E. coli* ATCC 25922 (18–34%), and *P. aeruginosa* ATCC 27853 (16–26%), respectively.

**Figure 8:**
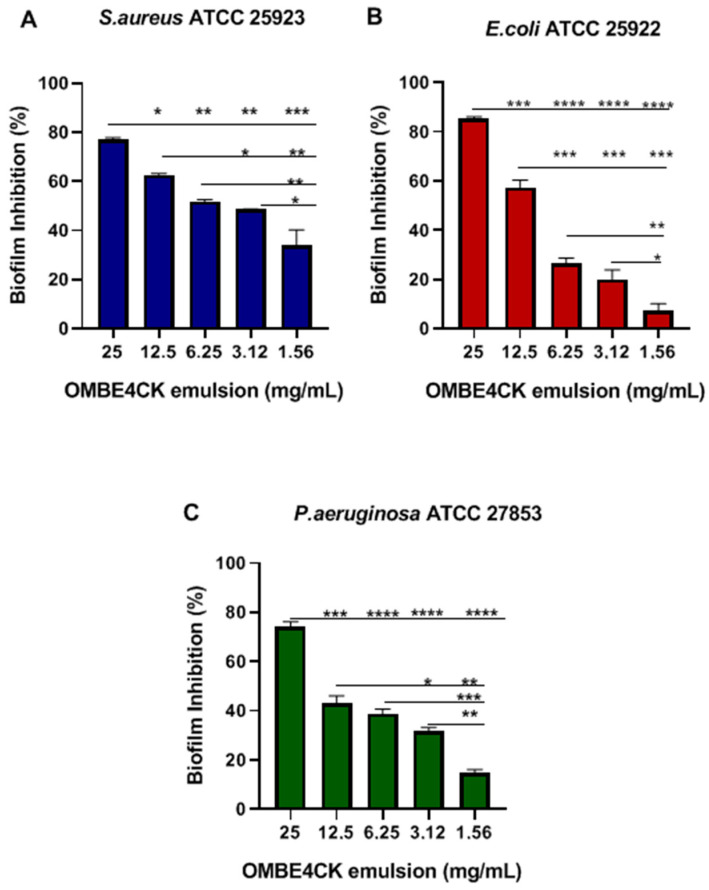
OMBE4CK inhibitory activity on biofilm formation by microorganisms. (**A**) *S. aureus* ATCC 25923; (**B**) *E. coli* ATCC 25922; (**C**) *P. aeruginosa* ATCC 27853. Values are the mean ± standard deviation (SD) of three independent experiments. * (*p* < 0.05), ** (*p* < 0.001), *** (*p* < 0.001), **** (*p* < 0.0001)—a significant difference in relation to concentrations.

The ability of the emulsion (OMBE4CK) resulting from the combination of BE4CK bioemulsifier and melaleuca essential oil to reduce biofilm formation by *S. aureus* ATCC 25923, *E. coli* ATCC 25922, and *P. aeruginosa* ATCC 27853 was evaluated using microtiter plates of 96 wells (Figure 8A–C).

For both strains, the use of emulsification at all concentrations resulted in significant inhibition of biofilm formation (*p* < 0.05). A biofilm inhibition of 77% for *S. aureus* ATCC 25923 (Figure 8A), 85% for *E. coli* ATCC 25922 (Figure 8B), and 74% for *P. aeruginosa* ATCC 27853 (Figure 8C) was obtained using the concentration of 25 mg/mL (*p* < 0.001). The antibiofilm activity of the emulsion (1.56 mg/mL–25 mg/mL) gradually decreased at low concentrations (1.56 mg/mL and 3.12 mg/mL), resulting in an inhibitory effect of 34% and 49% for *S. aureus* ATCC 25923 (Figure 8A); 7% and 20% for *E. coli* ATCC 25922 (Figure 8B); and 15% and 31% for *P. aeruginosa* ATCC 27853 (Figure 8C), respectively. Based on these results, the emulsion produced a significant antibiofilm effect against both pathogens.

#### 2.8.2. Inhibition of Biofilm Formation in an Enteral Feeding Catheter

##### Quantitative Analysis

**Figure 9:**
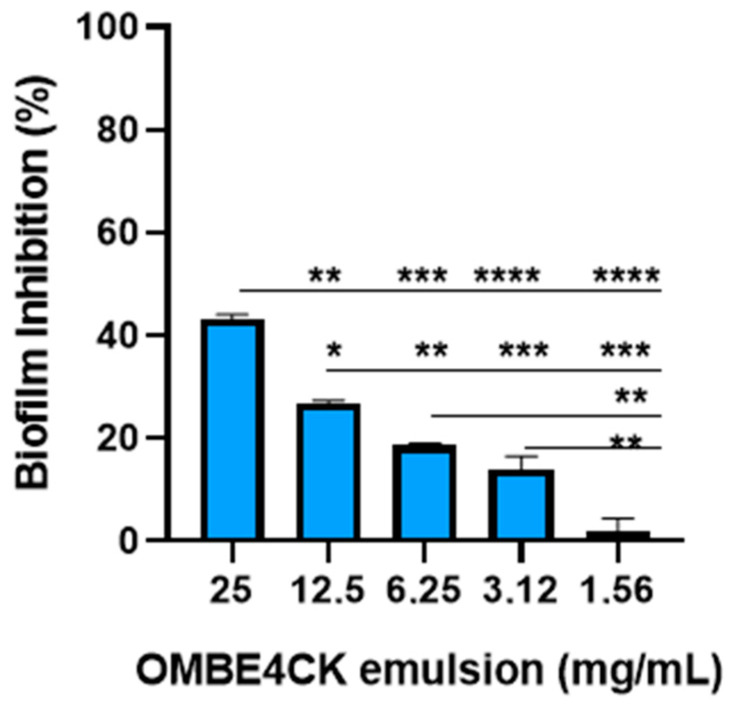
Inhibitory emulsion (OMBE4CK) activity on biofilm formation by *S. aureus* ATCC 25923 on the enteral feeding catheter surface. Values are mean ± standard deviation (SD) of three independent experiments. * *(p* < 0.05), **(*p* < 0.001), *** (*p* < 0.001), **** (*p* < 0.0001)—a significant difference in relation to concentrations.

The antibiofilm potential of the emulsion was determined by its ability to inhibit biofilm formation by *S. aureus* ATCC 25923 in an enteral feeding catheter, which corresponds to a polyurethane medical device (Figure 9). In the 25 mg/mL concentration, the emulsion showed a significant (*p* < 0.001) inhibitory potential of 43% for *S. aureus* ATCC 25923. While at lower concentrations (3.12 mg/mL–12.5 mg/mL), the antibiofilm activity of the emulsion gradually decreased, resulting in an inhibitory effect varying significantly (*p* < 0.05) between 14 and 27%. 

##### Semi-Quantitative Analysis

Through the semi-quantitative method of rolling catheters in a Petri dish containing TSA, catheter fragments exposed to the maximum concentration (25 mg/mL) of the OMBE4CK emulsion obtained a percentage of 4% contamination (with growth < 15 CFU/plate) by *S. aureus* ATCC 25923, whereas at the lowest concentrations (1.56 mg/mL–12.5 mg/mL), there was a range from 23% to 94% of colonization (with growth ≥ 15 CFU/plate) of *S. aureus* ATCC 25923 to polyurethane catheter surfaces (Table 3). At the concentration of 1.56 mg/mL of the emulsion, there was a 94% colonization of *S. aureus* ATCC 25923. Thus, biofilm inhibition depends on the emulsion concentration.

#### 2.8.3. Biofilm Eradication

**Figure 10:**
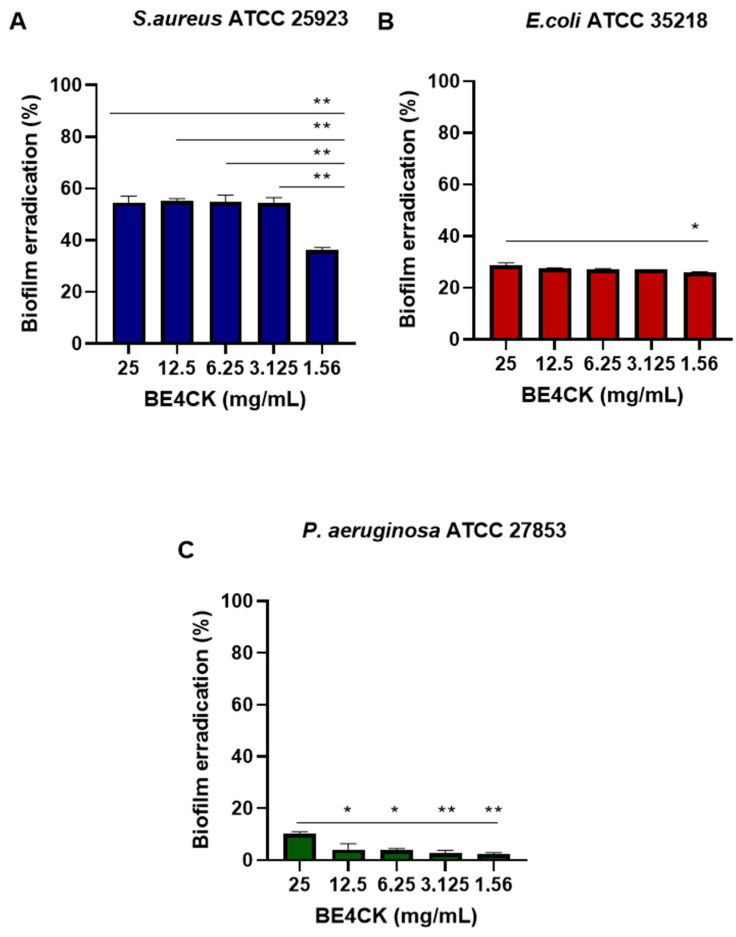
Eradication of the biofilm by the bioemulsifier BE4CK against microorganisms. Values are three independent experiments’ mean ± standard deviation (SD). * (*p* < 0.05), ** (*p* < 0.001)—a significant difference in relation to concentrations.

Biofilms formed by *S. aureus* ATCC 25923, *E. coli* ATCC 25922, and *P. aeruginosa* ATCC 27853 within 48 h and treated with the BE4CK were effectively disrupted at the highest concentration (25 mg/mL). At this concentration, the dispersion of biofilms formed by these microorganisms ranged from 10% to 55%, while at the lowest concentration (1.56 mg/mL), it ranged from 2% to 36% (Figure 10).

The biofilm formed by *S. aureus* ATCC 25923 was susceptible to the action of the BE4CK at all concentrations used (Figure 10A). At 25 mg/mL–3.12 mg/mL concentrations, the compound promoted eradication rates ranging from 54% to 55%. While at the lowest concentration (1.56 mg/mL), it removed 36% of the preformed biofilm. The increase in the BE4CK concentration did not promote significant differences in removing the *S. aureus* ATCC 25923 biofilm. Thus, eradicating the biofilm did not depend on the concentration of the BE4CK.

The action of BE4CK on the biofilm of *E. coli* ATCC 25922 and *P. aeruginosa* ATCC 27853 showed little effective results (Figure 10B,C). Preformed biofilms were significantly (*p* < 0.05) eradicated at concentrations of 1.56 mg/mL to 25 mg/mL of BE4CK, with biofilm reduction ranges from 29 to 26% *E. coli* ATCC 25922 (Figure 10B) and 2 to 10% for *P. aeruginosa* ATCC 27853 (Figure 10C).

**Figure 11:**
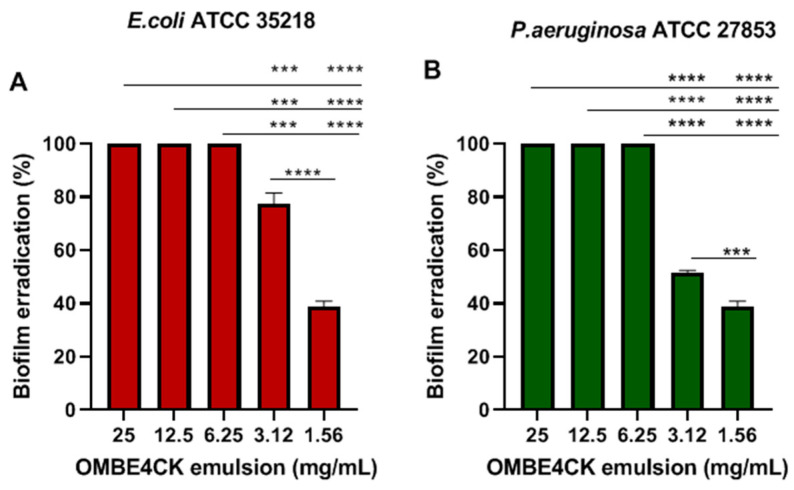
Biofilm eradication by OMBE4CK (emulsion) against microorganisms. (**A**) *E. coli* ATCC 25922, (**B**) *P. aeruginosa* ATCC 27853. Values are three independent experiments’ mean ± standard deviation (SD). *** (*p* < 0.0001), **** (*p* < 0.0001)—a significant difference in concentrations.

The emulsion OMBE4CK promoted significant dispersion (*p* < 0.0001) of the biofilm in all microorganisms tested (Figure 11A,B). The biofilms formed by *S. aureus* ATCC 25923 were utterly (100%) eradicated at all concentrations (1.56 mg/mL to 25 mg/mL) of the emulsion with no apparent dose–effect relationship between concentrations. The preformed biofilm by *E. coli* ATCC 25922 and *P. aeruginosa* 27853 was removed entirely (*p* < 0.0001) only at concentrations of 6.25 mg/mL–25 mg/mL, whereas at concentrations of 1.56 mg/mL and 3.12%, biofilm removal ranged from 39% to 77% for *E. coli* ATCC 25922 (Figure 11A) and 39% to 52% for *P. aeruginosa* 27853 (Figure 11B).

## 3. Discussion

### 3.1. Production and Characterization of the Bioemulsifier Produced by Candida krusei M4CK

In the present study, the yeasts were identified as belonging to *Candida metapsilosis* and *Candida krusei* species. Yeast is widely present in the environment, and many colonize soils, foods, leaf surfaces, and flowers [15]. 

*Candida krusei* is an ascomycetic yeast and is widely distributed in nature. In addition, it is frequently identified in cocoa fermentation in different places [16]. Despite being the same yeast, publications related to industrial applications often use the species names *Pichia kudriavzevii*, *Candida glycerinogenes,* and *Issatchenkia orientalis* in preference to *C. krusei* due to the negative safety connotations of using a pathogen in a biotechnological or food context [17]. 

In this study, the yeasts were isolated from the fruit *B. crassifolia*, popularly known as murici; this fruit originates from Central and South America and has been used for food production and folk medicine [18]. The isolation of yeasts with the potential to produce surface-active compounds indicates that the producers of SACs are widely distributed on the surfaces, internally found in the pulp of various fruits and plants, and have the potential for use in emulsifying systems and different biotechnological processes [19,20].

The *C. krusei* M4CK strain had better growth and performance in producing SACs with glycerol as a carbon and energy source. This discrepancy may be related to the type of carbon sources since these compounds can act in different ways, such as altering the structure of the biosurfactants and bioemulsifiers produced and, consequently, their emulsifying properties [21].

As the type of carbon source also plays a critical role in the production of surfactant compounds by microorganisms [22], it appears that the absorption of glycerol can help to optimize the production processes of biosurfactant glycolipids, such as the production of sophorolipids (SL) by *Starmerella bombicola* [23]. Like the present study, glycerol was reported as one of the best carbon sources for glycolipid production by *Pseudomonas* sp. [24], *a substrate suitable for* higher production and maximum emulsifying activity of the bioemulsifier produced by *Bacillus* sp. [25].

The bioemulsifier of *C. krusei* M4CK was called BE4CK. It showed an emulsifying activity equal to or greater than 50% in olive oil, sunflower, after frying, and hydrocarbons such as kerosene, hexane, and hexadecane. The best emulsifying activities of SACs were demonstrated in the presence of hydrocarbons such as kerosene, hexane, and hexadecane, in which an E_24_ ≥ 63.6% was reached. According to Willumsen and Karlson [26], E_24_ values equal to or greater than 50% are considered significant, and for Bosch et al. [27], an emulsion with this value is considered stable.

BE4CK can be classified as an amphiphilic biopolymer that contains monosaccharides (mannose and glucose) and long-chain aliphatic groups, such as 9-octadecenoic acid and hexadecanoic acid, in its polymer structure and is effective in emulsifying kerosene. The emulsification for BE4CK can be achieved through the interaction of hydrophobic (fatty acids) with hydrocarbons and the interaction of hydrophilics (monosaccharides) with water molecules. Furthermore, as it is a polymer, there is a greater probability of interaction due to the stearic phenomenon, which can lead to a more significant layer of emulsification of petroleum-derived hydrocarbons than vegetable oils. It is believed that there is a more outstanding balance of forces that stabilize kerosene emulsions, providing a higher emulsified layer [28]. Similar to the presented results, sophorolipids synthesized by yeasts of the genus *Meyerozyma* sp. were able to emulsify in hydrocarbons (kerosene, hexane, and hexadecane) and olive oil, and crude oil, the emulsifying activity for these compounds was more significant than 60% [28]. 

The bioemulsifier BE4CK showed a surface tension of the water of 44 mN/m. Other SACs, such as the glycolipid type produced by *Rhodotorula mucilaginosa* LBP5, reduced the surface tension of water to 40.7 mN/m [29]. However, it is observed that the surface tension of SACs produced by yeasts can be as low as 40 mN/m, followed by the glycolipid-type biosurfactant produced by *Rhodotorula* sp.CC01, whose critical micellar concentration tests indicated a value of 33.39 mN/m [30].

A surface tension reduction value similar to that obtained in the assay using BE4CK was observed for *Candida tropicalis* cultivated in n-hexadecane; this yeast produced an extracellular emulsifier that was able to reduce the surface tension of water from 70 mN/m to 49.5 mN/m. The same compound emulsified several hydrocarbons, including aromatic hydrocarbons [31]. Other SAC-producing yeasts have been identified, such as *Meyerozyma caribbica, Yarrowia lipolytica*, and *Trichosporon mycotoxinivorans* which, despite producing potent bioemulsifiers with high emulsification values in hydrocarbons, did not reduce the surface tension of water when tested [22,32,33].

E_24_ follows exponential growth for *C. krusei* M4CK from the growth phase up to 144 h, obtaining a higher emulsifying activity between 60 h and 100 h in the stationary phase. These data showed a linear correlation between possible cell concentration and emulsifying activity. This behavior can be explained by the fact that the emulsifier is being produced to improve the assimilation of the carbon source. In the production associated with growth, there are parallel relationships between growth, substrate utilization, and the production of surface-active compounds [22,34,35]. Bioemulsifier production kinetics partially associated with cell growth have also been reported for yeast species *Geotrichum* sp. and *Trichosporoon montevideense* [6].

Bioemulsifier effectiveness depends on emulsifying activity over various temperatures, pH, and salinity [36]. The bioemulsifier BE4CK maintained stable emulsions over a wide temperature range (120 °C) for sources of olive and sunflower oil (E_24_ ≥ 50%) and hydrocarbons (E_24_ ≥ 63%) such as kerosene, hexane, and hexadecane. However, for the frying oil (E_24_ of 40%), there was a 10% reduction in the emulsifying activity. Such results demonstrate that BE4CK is thermostable.

The stability of the bioemulsifier produced by *C. lipolytica* at high temperatures has also been reported at temperatures up to 100 °C [36]. Likewise, biosurfactant sophorolipids produced by *Candida keroseneae* showed high thermal stability with a maximum emulsification activity of 60% in kerosene and preserved 97.25% of its original activity at 120 °C [37]. The reduction in emulsifying activity may be related to the denaturation of the protein component of the bioemulsifier during heat treatment. This property depends on the molecule’s chemical structure, as many emulsifiers are stable in significant temperature variations or show minor deviations in higher temperatures [22].

The stability of emulsification by BE4CK was maintained for sunflower and olive oil sources only at acidic pH (3–6). At the same time, BE4CK maintained emulsifier stability in kerosene in all pH ranges (3–9). The inhibition of oil activity and the pronounced reduction in kerosene were recorded at pH values above 6.0. The decrease in emulsifying activity is likely related to structural changes in bioemulsifiers caused by extreme pH conditions [33,38]. In addition to maintaining emulsifying activity at high temperatures and pH ranges, the bioemulsifier BE4CK carried the E_24_ in different concentrations of MgCl_2_, NaCl, and CaCl_2_. Due to its nature, the amphiphilic biopolymer BE4CK appears to be more stable and less susceptible to the action of ions for coalesce activity. Similar results to the present study were demonstrated for the bioemulsifier produced by *Pseudomonas stutzeri* NJtech 11-1. The crude bioemulsifier can tolerate 50% NaCl, MgCl_2_, and CaCl_2_ with only a 29.8% loss of E_24_. *Meyerozyma caribbica* yeasts also produced stable bioemulsifiers at high salinity levels [39]. In contrast, a bioemulsifier produced by *Candida lipolytica* using burned motor oil as a hydrophobic substrate showed that its emulsifying stability at 10% NaCl concentration was inactive [36].

By the toxicity test, the bioemulsifier showed no toxicity at the concentrations. Cells remained viable and did not undergo cell death; cell viability was ≥88.89%. Toxicity tests with SACs of biosurfactants produced from species of the genus *Candida* also indicated the absence of toxicity of these biomolecules against marine bioindicators [40]. Our results were in accordance with previous work by [41], who reported no risk of toxicity bioemulsifier produced by *S*. *cerevisiae* against normal epithelial kidney cells at concentrations up to 250 µg/mL. However, further, in vivo tests are required to confirm the safety of bioemulsifier BE4CK.

### 3.2. Antibacterial and Antibiofilm Activity of the C. krusei M4CK Bioemulsifier with Melaleuca Essential Oil

When evaluating the antimicrobial activity through the diffusion assay, it was verified that the isolates of *E. coli* ATCC 25922, *Enterococcus faecalis* ATCC 35218, *P. aeruginosa* ATCC 27853, and *S. aureus* ATCC 25923 did not show sensitivity to the bioemulsifier BE4CK. However, when combined with melaleuca essential oil emulsion (OMBE4CK), they exhibited antimicrobial activity for *E. coli* ATCC 25922 isolates, *E. faecalis* ATCC 35218, and *S. aureus* ATCC 25923. The antimicrobial activity of the OMBE4CK emulsion was more efficient for *E. coli* ATCC 25922 and did not cause sensitivity in *P. aeruginosa* ATCC 27853. These data indicate that melaleuca essential oil can be a candidate for producing formulations with surface-active compounds synthesized by microorganisms.

Antimicrobial performance similar to the bioemulsifier BE4CK was demonstrated for sophorolipid produced by non-pathogenic yeasts of *Candida bombicola* (ATCC 22214); this biosurfactant could not inhibit the growth of *E. coli* alone [42]. But, in combination with antibiotics, it promoted a total inhibition of the development of the microorganism. This ability is not only related to the inhibitory effect increased by the additive action of two antimicrobial agents; it may also be associated with the performance of SL to facilitate the entry of molecules into bacterial cells [42]. So, the antimicrobial effect of biosurfactants is due to their potential to form pores within cell membranes; this feature can increase the effectiveness of antimicrobial agents [43].

Antimicrobial resistance to antibiotics is often related to the ability of microorganisms to form biofilms, which exhibit dozens of attributes within this structure, making them difficult to eliminate [44]. Given this fact and the antimicrobial capacity of the emulsion (BE4CK and OMBE4CK), the action of the bioemulsifier and the emulsion in the inhibition and eradication of biofilms formed by *E coli* ATCC 25922, *P. aeruginosa* ATCC 27853, and *S. aureus* ATCC 25923.

The compound showed a concentration-dependent effect, with the best biofilm formation inhibitory activity against bacterial pathogens. Higher BE4CK activity was found in. *S. aureus* ATCC 25923, while less activity was demonstrated for *P. aeruginosa* ATCC 27853. The BE4CK activity profile may be related to the cell wall characteristics of both bacteria. 

The antibiofilm activity of the emulsion gradually decreased at lower concentrations. Melaleuca essential oil has already demonstrated antibiofilm activity against *S*. *aureus* and *E. coli* [45,46]. On the other hand, there is previous evidence of the synergism between SACs and essential oils; this fact reinforces the hypothesis of the ability of SACs to facilitate the entry of antimicrobial molecules into cells [47]. In addition, melaleuca essential oil emulsions prove to be an excellent microbial control strategy, as their improved tissue infiltration capacity and ability to stabilize the volatilization of terpenes, their main antimicrobial compounds, are considered. In this study, tea tree oil emulsions (OMBE4CK) were stable up to 250 (E_250_) evaluation days, which enhances their use in formulations.

Biofilm formation on medical devices is one of the leading causes of nosocomial infections and represents a severe public health problem. As a possible alternative for solving this problem, the potential of the emulsion was also evaluated for inhibiting biofilm formation in polyurethane catheters at different concentrations. At the highest concentration (25 mg/mL), OMBE4CK showed a significant % inhibitory potential of 43% for *S. aureus* ATCC 25923. Other authors also reported this ability to interfere with biofilm formation. Mendes et al. [48] showed that a mixture of sophorolipids produced by *Starmerella bombicola* CBS 6009 with sodium dodecyl sulfate (SDS, commercial surfactant) demonstrated inhibitory activity of biofilm formation by *S. aureus* and *E. coli* [48]. Another study proposed by Pontes et al. [49] determined that the presence of sophorolipids on the surface of the catheter (silicone) decreased the hydrophobicity of the material and the formation of biofilm by *S. aureus* ATCC 25923 and *E. coli* ATCC 25922 [49].

The OMBE4CK emulsion showed better activity in removing biofilms compared to the isolated action of BE4CK. Biofilm eradication may be related to the bacteriostatic and bactericidal effects of the OMBE4CK emulsion.

Biofilms are microbial communities that are difficult to eliminate. The potential of the bioemulsifier BE4CK and the emulsion OMBE4CK in eradicating biofilms was evaluated to seek strategies to disperse this structure. The treatment with the bioemulsifier alone on the biofilm of *E. coli* ATCC 25922 and *P*. *aeruginosa* ATCC 27853 did not show effective results in all concentrations. In contrast, *S. aureus* ATCC 25923 biofilms were significantly eradicated.

The low dispersion of the biofilm of *E. coli* ATCC 25922 and *P. aeruginosa* ATCC 27853 can be attributed to the difficulty of the SACs in penetrating the biofilm matrix; in this sense, higher concentrations of SACs are necessary for the detachment of the preformed biofilm. A surface-active substance must penetrate the biofilm, and the substrate interface must detach from the biofilm [50]. After their penetration, they can change surface properties, leading to biofilms’ separation from surfaces [50].

In the complete eradication of the *S. aureus* biofilm, the pit is probably related to the ability of the *bioemulsifier BE4CK* to increase the action of melaleuca essential oil, facilitating its entry through the biofilm matrix. The biofilm matrix presents a dynamic to be considered when applying a particular antibiotic compound. In this case, the action to dispose of the cells can be improved using SACs that alter the surface characteristics, such as hydrophobicity or hydrophilicity, in addition to the electrical charge characteristics (repulsion and attraction) [51]. Emulsified (containing essential oils) systems can be interesting in controlling microbial biofilms. The present study observed that the emulsion at higher concentrations was more effective than the bioemulsifier BE4CK in eradicating the *P. aeruginosa* biofilm. 

## 4. Materials and Methods

### 4.1. Yeast Isolation

For the isolation and selection of yeasts of interest, fruits of the species *Anacardium occidentale*, *Byrsonima crassifolia*, *Citrus reticulata*, and *Platonia insignis* were collected from plants present in sites located in the Municipalities of São José de Ribamar and São Luís, Maranhão. The fruits selected for yeast isolation were in their intermediate stage of maturation. The harvested fruits were stored in thermal boxes with ice and transported to the Applied Microbiology Laboratory of CEUMA University for further processing.

The fruits were initially sanitized with 5% sodium hypochlorite to isolate SAC-producing yeasts and washed three times with sterile distilled water to remove sodium hypochlorite. Subsequently, approximately 20 g of fruit samples were inoculated into Erlenmeyer flasks with the addition of a minimal mineral medium (MM) containing (in g/L) 1.0 g K_2_HPO_4_, 1.0 g KH_2_PO_4_, 0.2 g MgSO_4_·7H_2_O, 1.0 g NH_4_NO_3_, 0.002 g CaCl_2_·2H_2_O, and 0.2% yeast extract with 2% sunflower oil or glycerol added as the only carbon source. The final pH of the medium was 7.2.

The flasks were incubated at 30 °C with agitation at 140 rpm for seven days. After incubation, 1 mL of the cultures were serially diluted in a sterile saline solution (0.85% NaCl). Aliquots of 50 µL of decimal dilutions were spread on the surface of the Sabouraud Dextrose Agar medium (SDA, Difco TM, Detroit, MI, USA) to isolate, quantify, and characterize colonies.

Samples of yeast colonies were inoculated in the SDA medium to obtain a pure culture; later, microcultures were performed for morphological characterization. The yeasts were kept in inclined tubes with the SDA medium and stored in cryotubes at –80 °C in Sabouraud broth (Difco) with 20% glycerol.

### 4.2. Screening and Identification of SAC-Producing Yeasts

#### 4.2.1. Characterization of Emulsifying Activity (E_24_) of Culture Supernatant

The yeast isolates were inoculated at an initial concentration of 0.1 OD_600_ nm (≈1 × 10^7^ CFU/mL) in Erlenmeyer flasks with 50 mL of a minimal mineral medium (MM) containing (in g/L) 1.0 g K_2_HPO_4_, 1.0 g KH_2_PO_4_, 0.2 g MgSO_4_·7H_2_O, 1.0 g NH_4_NO_3_, 0.002 g CaCl_2_·2H_2_O, and 0.2% yeast extract with the addition of 2% glycerol or 2% sunflower oil (as carbon sources for SAC production) as proposed by Monteiro et al. [6], with modifications. Each yeast isolate was incubated on the carbon sources to evaluate the production of SACs. The microbial cultures were incubated for up to 144 h at 30 °C under agitation at 140 rpm. After incubation, the cultures were centrifuged at 5000× *g* for 10 min, and the supernatants were used in the emulsifying activity (E_24_) test. The cell-free supernatant’s surface tension (TS) was determined using a Du Nouy ring-type tensiometer (Manual Tensiometer, model k6, Krüss GmbH, Hamburg, Germany). The TS measurement was performed at 20 °C with modifications). The microbial cultures were performed in duplicate.

The emulsifying activity was determined by the emulsification index (E_24_), in which the supernatant was applied directly into test tubes in the proportion of 50% (2.5 mL) and 50% (2.5 mL) of different hydrophobic substrates (olive oil, sunflower oil, and fried foods, kerosene, hexane, and hexadecane). The mixture was subjected to vortex agitation for 2 min. After 24 h, the emulsification activity (E_24_) was determined by dividing the height of the emulsified layer by the total size of the mixture, expressed as a percentage [52]. For tests, rhamnolipids (100 mg/mL) (Di-rhamnolipid, Sigma-Aldrich, St. Louis, MO, USA) were a positive control and the MM medium was a negative control. The emulsifying activity tests were performed in triplicate.

MALDI-TOF MS-based identification of yeast isolates was performed according to Bruker Daltonics (Bremen, Germany) using the protocol in Aslani et al. [53].

#### 4.2.2. Determination of the Surface Tension of Water in the Presence of a Bioemulsifier

Surface tension (TS) was measured using a Du Nouy ring-type tensiometer (Manual Tensiometer, model k6, Krüss GmbH, Hamburg, Germany). The TS measurement was performed at 20 °C with modifications. For tests, sodium dodecyl sulfate (SDS, 100 mg/L) was the positive control and the MM medium was the negative control. The TS measurement was performed at 20 °C with modifications. The concentration of the bioemulsifier used varied from 0.01 mg/mL to 10 mg/mL. The round hanging from the scale’s hook was immersed in the liquid. Then, the call was slowly pulled upwards, lowering the sample plate. Finally, the force applied to the ring during traction across the surface was recorded. The measurement was repeated three times; the platinum ring was washed three times with distilled water and three times with alcohol between measurements [6]. Measurement of the surface tension of the distilled water was used as a control.

### 4.3. Chemical Composition of SACs

Standard methods were used to determine the chemical composition of the bioemulsifier. The carbohydrate content was determined by the phenol–sulfuric acid method of Dubois et al. [54] using D-glucose as a standard. Protein content was measured as described by Bradford et al. [55] using bovine serum albumin as a standard. Lipid content was estimated using the K117—BIOCLIN monoreagent triglyceride kit. All experiments were performed in triplicate. The carbohydrate and lipid composition of the bioemulsifier (SAC) was determined by gas chromatography and mass spectrometry using the previously (GC-MS) developed protocol [6].

### 4.4. Growth Kinetics

The inoculum was prepared from *C. krusei* cells incubated in a solid Sabouraud medium for 24 h at 32 °C and washed three times with saline solution (NaCl 0.85%, *w*/*v*). After washing, the cells were resuspended in 50 mL plastic tubes containing 20 mL of the MM medium. Optical density readings at 600 nm were performed. The values obtained determined the volume to be transferred to the test flasks for a final concentration of 0.02 OD, corresponding to (≈5 × 10^6^ CFU/mL). The experiments were conducted in 500 mL Erlenmeyer flasks containing 200 mL of the MM medium added with 2% glycerol. The flasks were incubated at 32 °C under continuous agitation at 180 rpm for up to 144 h. The samples were removed at 12 h or 24 h to determine cell concentration and emulsifying activity. The tests were performed in three repetitions.

Cell growth was determined by removing the aliquots from the culture medium and obtaining decimal dilutions from which samples were removed, serially diluted, and plated in a solid Sabouraud medium to determine CFU/mL. The values obtained were converted into Log_10_ of CFU/mL [6]. The emulsification activity (E_24_) described in Section 4.2.1 also evaluated the supernatant samples for emulsifying activity. The tests were performed in three repetitions.

### 4.5. Stability of the Bioemulsifier in Different NaCl, MgCl_2_, and CaCl_2_ Concentrations, Autoclavings, and pH Variations

The effects of different concentrations (10, 30, and 50%) of magnesium chloride (MgCl_2_), sodium chloride (NaCl), and calcium chloride (CaCl_2_), a temperature of 121 °C for 15 min (autoclaving), and different pH values (3–10) were analyzed in the activity of the bioemulsifier BE4CK. The emulsification activity (E_24_) was evaluated after adjusting the pH minimum medium with sodium hydroxide (NaOH) or hydrochloric acid (HCl). 

### 4.6. Cytotoxicity Test

The assay for evaluation of bioemulsifier cytotoxicity was performed using African green monkey kidney cells (VERO, ATCC), according to Sieuwerts et al. [56]. For the assay, the cells were washed with phosphate-buffered saline and resuspended in Dulbecco’s Modified Eagle Medium (DMEM, Thermo Scientific, Erlangen, Germany) culture medium supplemented with 10% fetal bovine serum (FBS) with 100 IU/mL of penicillin and 100 µg/mL of streptomycin. Cell counting was performed to determine the cell number with an equal volume of trypan blue. Briefly, 100 µL of DMEM with 10% of FBS was added to the 96-well plate (TPP, Trasadingen, Switzerland). Cells were added to the wells (1.5 × 10^6^ cells per well) and treated with 5000 µg/mL–2 µg/mL diluted in DMEM and PBS for the negative control. The plates were incubated for 24 h at 37 °C, 5% CO_2_, and a 90% humidity incubator. After incubation, 20 µL of MTT (3-4,5-dimethylthiazol-2,5-diphenyltetrazolium bromide, Sigma-Aldrich) at 5 mg/mL was added into each well in the 96-well plate and incubated for four hours in 37 °C, 5% CO_2,_ and a 90% humidity incubator. After the incubation, 170 µL of the medium with MTT was removed from every well, and 100 µL DMSO (Sigma-Aldrich, USA) was added to each well to extract and solubilize the formazan crystal. Finally, the plate was read at a 570 nm wavelength using TP-Reader Plus (ThermoPlate, China). Each extract concentration (flavonoids) was assayed in triplicate in two independent experiments. The following formula calculated the percentage of proliferation: [OD sample − OD control] × 100% proliferation = OD control.

### 4.7. Well Diffusion Test in Agar

The tests were performed according to Johnson et al. [57] as modifications for the study of the antimicrobial activity of the SAC. For *Escherichia coli* ATCC 25922, *Enterococcus faecalis* ATCC 35218, *Pseudomonas aeruginosa* ATCC 27853, and *Staphylococcus aureus* ATCC 25923 were added in a Tryptic Soy Agar (TSA, Difco TM, Detroit, MI, USA) for 24 h at 30–37 °C. Subsequently, the isolates were suspended in 0.9% NaCl and adjusted to equal the standard turbidity to 0.5 on the McFarland scale (1.5 × 10^8^ CFU/mL). The suspensions were used for seeding in the TSA medium stabilized at 37 °C (0.1 mL microorganism test suspension/10 mL medium). The TSA culture medium was poured into 90 mm Petri dishes (20 mL medium/dish) and allowed to solidify. Once the medium was solid, four 9 mm diameter wells were made with sterilized plastic straws and filled with one volume of SACs produced by *C. krusei*, melaleuca essential oil, and emulsion (a mixture of SACs and essential oil melaleuca). Plates were pre-incubated for one hour at room temperature to ensure adequate diffusion and finally incubated at 30–37 °C for 24 h. Inhibition halos were evaluated in mm. All tests were performed in triplicate.

### 4.8. Antibiofilm Activity

#### 4.8.1. Inhibition of Biofilm Formation on Polystyrene

Suspensions of biofilm-forming isolates were prepared according to the methodology established by Merghini et al. [58], with modifications. For the tests, breaks of each isolate of *E. coli* ATCC 25922, *P. aeruginosa* ATCC 27853, and *S. aureus* ATCC 25923 were prepared in Mueller–Hinton broth (Difco TM, Detroit, MI, USA) plus 5% sucrose at a cell concentration of 0.5 on the McFarland turbidity scale (1.5 × 10^8^ CFU/mL). The bioemulsifiers were diluted in Mueller–Hinton broth at 25 mg/mL, 12.5 mg/mL, 6.25 mg/mL, 3.12 mg/mL, and 1.56 mg/mL, with a final volume of 200 µL. Each well containing the bioemulsifier received 20 µL of the standardized bacterial suspension. The plate was then incubated for 24 h at 35 ± 2 °C.

The contents of each well were aspirated, the wells were gently washed three times with 200 μL of *phosphate-buffered saline* (PBS, pH 7.4), and the plates were kept at room temperature to dry. The adhered biomass was fixed with 200 μL of methanol (P.A 99% *v*/*v*) for 10 min. Then, 200 μL of a 0.5% crystal violet solution was added to each well. Subsequently, the crystal violet was removed, and the wells were washed three times with distilled water. Then, 200 µL of alcohol 95% (*v*/*v*) was added to each well, and the OD of the crystal violet extract was determined in a microplate reader at 570 nm. Using the formula below, the percentage of biofilm inhibition was calculated.

Percentage inhibition (%) = 100 − ((OD 570 nm of the treatment)/(OD 570 nm of the control) × 100).

This study also evaluated the activity of the emulsion resulting from the combination of the bioemulsifier and melaleuca essential oil (OMBE4CK) in inhibiting biofilm formation for each bacterium. For this, the same methodological procedure was followed. The used concentrations of OMBE4CK were 25 mg/mL to 1.56 mg/mL in emulsion.

To form the emulsion between the SAC and the melaleuca essential oil (commercially purchased), 50 mg of BE4CK (SAC) was used. This amount was dissolved in 10 mL of water for sterile injection; later, this solution was applied directly in test tubes in the proportion (2 mL) of the solution and (2 mL) the melaleuca essential oil. The mixture was subjected to vortexing for 2 min, and the emulsion formed was used in the tests at different concentrations (1.56 mg/mL–25 mg/mL).

#### 4.8.2. Inhibition of Biofilm Formation in an Enteral Feeding Catheter

##### Quantitative Analysis

To evaluate the inhibition of biofilm formation in a polyurethane catheter (enteral feeding catheter, Embramed SP, Brazil), sterile segments of the device, measuring approximately 4 cm, were immersed in a TSB medium containing 0.5 × 10^5^ CFU/mL of *S. aureus* 25923 suspensions and OMBE4CK (emulsion) concentrations (1.56 mg/mL–25 mg/mL). Catheter fragments were incubated for 24 h at 37 °C. After incubation, the catheters were washed with 200 µL of sterile saline three times and vortexed for 2 min. About 100 µL of the last wash was removed for serial dilutions in sterile saline. After the decimal dilutions, 30 µL of the sample were transferred to the surface of Petri plates containing the TSA medium, and the samples were homogenized with Drigalsky loops. After drying the samples, the plates were incubated for 24 h at 37 °C. After incubation, the colony-forming unit (CFU/mL) count was performed. All tests were performed in triplicate.

A semi-quantitative analysis of the inhibition of biofilm formation on the surface of a polyurethane catheter was performed by rolling the catheter segment in the Petri dish containing the TSA medium (Difco TM, Detroit, MI, USA). The semiquantitative technique distinguishes colonization (greater than or equal to 15 colonies) from contamination [59].

#### 4.8.3. Biofilm Eradication

An evaluation of the influence of the bioemulsifier BE4CK on biofilms produced by *E. coli* ATCC 25922, *P. aeruginosa* ATCC 27853, and *S. aureus* ATCC 25923 were performed after the formation of microbial biofilms on the surface of the wells of 96-well polystyrene plates according to the established methodology by Morais et al. [60], with modifications. A total of 200 µL of Brain Heart Infusion (BHI, Difco TM, Detroit, MI, USA) with 2% sucrose were added to all wells; then, 10 µL of standardized bacterial suspension on the McFarland scale (~0.5 × 10^5^ CFU/mL) was added, the positive control was performed with the addition of the BHI medium + sucrose, and 10 µL of bacterial suspension was added. For the negative control, only the BHI medium with sucrose was added. The plates were incubated for 48 h at 37 °C. After incubation, the wells were gently washed with 200 µL of sterile saline three times in each well; then, 200 µL of concentrations of 25 mg/mL were added; 12.5 mg/mL; 6.25 mg/mL; 3.12 mg/mL; and 1.56 mg/mL, which were incubated for 24 h. After incubation, washing was performed with 100 µL of a saline solution (NaCl, 0.85%). Subsequently, 100 µL of saline was added to the plate wells, and homogenization was carried out. A total of 50 µL was taken from the well, and decimal dilutions were performed for plating the samples. Viable cells were determined using microdroplet plating, for which approximately 10 µL were transferred to a Tryptic Soy Agar (TSA, Difco TM, Detroit, MI, USA) plate as a drop; after drying, the dish was incubated correctly for 24 h in the bacteriological oven. After incubation of the plates, the colony-forming unit (CFU/mL) count was performed. 

This study also evaluated the activity of the emulsion resulting from the interaction between the bioemulsifier and melaleuca essential oil (OMBE4CK) in eradicating the biofilm for each isolate. For this, the same methodological procedure was followed. The used concentrations of OMBE4CK were 25 mg/mL, 12.5 mg/mL, 6.25 mg/mL, 3.125 mg/mL, and 1.56 mg/mL of the emulsion. All tests were performed in triplicate.

### 4.9. Statistical Analysis

Data were submitted for statistical analysis using the one-way ANOVA procedure in GraphPad Prism (version 8.0.2). All duplicate results are expressed as mean ± standard deviation. Differences were examined using the Tukey test, with a significance level of 95%. The significance levels were set at *p* < 0.05, *p* < 0.001, and *p* < 0.0001.

## 5. Conclusions

This study presented a bioemulsifier derived from *Candida krusei* M4CK isolated from fruit murici and showed an excellent ability to use glycerol as a substrate for growth and bioemulsifier production. This biomolecule showed promising properties in emulsifying different sources of vegetable oil and hydrocarbons, a lack of toxicity, and stability of emulsions in a wide range of temperatures, pH, and salinity. Furthermore, the bioemulsifier BE4CK and its combination with melaleuca essential oil (OMBE4CK) inhibited bacterial biofilm formation and eradicated bacterial biofilm on polystyrene surfaces and catheters. Such properties emphasize that the bioemulsifier can represent a new source for biomedical development in emulsion formulations containing essential oils and can be a strategy for reducing the colonization of medical devices and surface abiotics.

## Figures and Tables

**Table 1:** Yeasts isolated from fruits and substrate as the sole carbon source for growth.

Fruit	Popular Name	Identification	Substrate
*Anacardium occidentale*	Cashew	*Candida krusei* C1CK	Glycerol
*Anacardium occidentale*	Cashew	*Candida krusei* C2CK	Glycerol
*Byrsonima crassifolia*	Murici	*Candida krusei* M1CK	Glycerol
*Byrsonima crassifolia*	Murici	*Candida krusei* M2CK	Glycerol
*Byrsonima crassifolia*	Murici	*Candida krusei* M3CK	Glycerol
*Byrsonima crassifolia*	Murici	*Candida krusei* M4CK	Glycerol
*Byrsonima crassifolia*	Murici	*Candida metapsilosis* M1CM	Sunflower oil
*Byrsonima crassifolia*	Murici	*Candida metapsilosis* M2CM	Sunflower oil
*Citrus reticulata*	Tangerine	*Candida metapsilosis* T2CM	Sunflower oil
*Platonia insignis*	Bacuri	*Candida metapsilosis* B1CM	Glycerol
*Platonia insignis*	Bacuri	*Candida metapsilosis* B2CM	Glycerol
*Platonia insignis*	Bacuri	*Candida metapsilosis* B3CM	Glycerol
*Platonia insignis*	Bacuri	*Candida metapsilosis* B4CM	Sunflower oil

**Table 2:** Antimicrobial activity of the melaleuca essential oil, bioemulsifier alone, and the combination of BE4CK and OMBE4CK (emulsion) against microorganisms.

Compound	Antimicrobial Activity (mm)			
	*E. coli* ATCC 25922	*E. faecalis* ATCC 35218	*P. aeruginosa* ATCC 27853	*S. aureus* ATCC 25923
BE4CK (25 mg/mL)	N	N	N	N
Melaleuca essential oil	28.5 ± 2.1	26 ± 1.4	17 ± 2.8	33.5 ± 0.7
OMBE4CK	34.5 ± 6.3	14.5 ± 2.1	-	24 ± 1.4

N—no antimicrobial activity. Values are three independent experiments’ mean ± standard deviation (SD). (*p* < 0.05)—a significant difference in relation to concentrations.

**Table 3:** Quantitative CFU/mL of *S. aureus* ATCC 25923 on the catheter surface.

Emulsion Concentration (mg/mL)	CFU/Plate		Classification
	N°	%	
25	4	14	Contamination
12.5	71	23%	Colonization
6.25	83	26%	Colonization
3.12	213	68%	Colonization
1.56	295	94%	Colonization
Control	314	100%	Colonization

CFU/mL: colony-forming unit per milliliter.

## Data Availability

Not applicable.

## Supplementary Materials

The following supporting information can be downloaded at https://www.mdpi.com/article/10.3390/antibiotics12121686/s1, Table S1. E24 values from yeasts from *Anacardium occidentale*, *Byrsonima crassifolia*, *Citrus reticulata*, and *Platonia insignis* fruits, Table S2. Determination of final surface tension (mN/m) in medium after cell growth yeasts strains. Figure S1. Antimicrobial activity of BE4CK and emulsion (OE Melaleuca and BE4CK- OMBE4CK) by agar diffusion against *E. coli* ATCC 25922.
