# Supplementary material for: Candida krusei M4CK Produces a Bioemulsifier That Acts on Melaleuca Essential Oil and Aids in Its Antibacterial and Antibiofilm Activity
Source: Antibiotics (Basel). 2023 Nov 30;12(12):1686. doi: 10.3390/antibiotics12121686 (PMC10740703; doi:10.3390/antibiotics12121686)
Supplement: Supplementary file 1 [file antibiotics-12-01686-s001.zip › Supplementary Table S1.pdf]

Supplementary Table S1. E_{24} values from yeasts from A*nacardium occidentale*, *Byrsonima crassifolia*, *Citrus reticulata,* and *Platonia insignis* fruits.

**Substrates**	**E_{24}**												
	C1CK	C2CK	M1CK	M2CK	M3CK	M4CK	M1CM	M2CM	T2CM	B1CM	B2CM	B3CM	B4CM
**sunflower oil**	50%	50%	N	55%	N	60.75%	N	N	N	N	N	N	N
**olive oil**	N	48%	N	56.75%	36%	61.25%	N	35%	N	N	50%	N	N
**frying oil**	N	50%	N	50%	33%	50%	N	33%	N	N	51%	N	N
**kerosene**	60%	51%	64%	N	46%	65.85%	40%	33%	37%	37%	N	N	N
**hexane**	60%	50%	37%	N	N	63%	N	N	33%	N	62,5%	N	N
**hexadecane**	48%	49%	32.5%	62.9%	N	61.7%	N	N	N	N	62%	N	N
