# Supplementary material for: Candida krusei M4CK Produces a Bioemulsifier That Acts on Melaleuca Essential Oil and Aids in Its Antibacterial and Antibiofilm Activity
Source: Antibiotics (Basel). 2023 Nov 30;12(12):1686. doi: 10.3390/antibiotics12121686 (PMC10740703; doi:10.3390/antibiotics12121686)
Supplement: Supplementary file 1 [file antibiotics-12-01686-s001.zip › Supplementary Table S2.pdf]

Supplementary Table S2. Determination of final surface tension (mN/m) in medium after cell growth yeasts strains

Yeast	substrate	Final surface tension in medium mN/m
Candida krusei C1CK	Glycerol	55
Candida krusei C2CK	Glycerol	55.2
Candida krusei M1CK	Glycerol	54.5
Candida krusei M2CK	Glycerol	55.2
Candida krusei M3CK	Glycerol	56.2
Candida krusei M4CK	Glycerol	44
Candida metapsilosis M1CM	sunflower oil	45
Candida metapsilosis M2CM	sunflower oil	46
Candida metapsilosis T2CM	sunflower oil	56.4
Candida metapsilosis B1CM	Glycerol	59.3
Candida metapsilosis B2CM	Glycerol	56
Candida metapsilosis B3CM	Glycerol	52
Candida metapsilosis B4CM	sunflower oil	62
