# Supplementary figures and images for: Candida krusei M4CK Produces a Bioemulsifier That Acts on Melaleuca Essential Oil and Aids in Its Antibacterial and Antibiofilm Activity
Source: Antibiotics (Basel). 2023 Nov 30;12(12):1686. doi: 10.3390/antibiotics12121686 (PMC10740703; doi:10.3390/antibiotics12121686)

**Melaleuca**

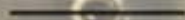

**28,5 mm**

**4CK**

**Melaleuca + 4CK**

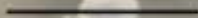

**30 mm**

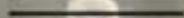

**39 mm**

Supplement: Supplementary file 1 [file antibiotics-12-01686-s001.zip › Supplementary Figure S1.pdf]
